# An Unusual Presentation of Cronkhite–Canada Syndrome With Hypothyroidism

**DOI:** 10.1155/carm/7336583

**Published:** 2025-09-10

**Authors:** Carlotta Crisciotti, Alessandra Marchese, Pasquale De Cata, Katerina Vjero, Claudia Vattiato, Vitantonio Caramia, Federico Biagi, Giovanni Arpa

**Affiliations:** ^1^Department of Internal Medicine and Therapeutics, University of Pavia, Pavia, Italy; ^2^Department of Internal Medicine, Istituti Clinici Scientifici Maugeri IRCCS, Pavia, Italy; ^3^Department of Digestive Endoscopy, Istituti Clinici Scientifici Maugeri IRCCS, Pavia, Italy; ^4^Gastroenterology Unit of Pavia Institute, Istituti Clinici Scientifici Maugeri IRCCS, Pavia, Italy; ^5^Anatomic Pathology Unit, Istituti Clinici Scientifici Maugeri IRCCS, Pavia, Italy

## Abstract

**Background:** Cronkhite–Canada syndrome (CCS) is a rare, nonhereditary gastrointestinal polyposis characterized by diffuse polyps, hyperpigmentation, onychodystrophy, and alopecia. Hypothyroidism has been infrequently reported in association with CCS.

**Case Presentation:** A Caucasian male in his late 70s presented with dysgeusia, asthenia, and significant weight loss, initially diagnosed with Crohn's disease. Physical examination showed onychodystrophy, hyperpigmentation, and leg edema. Laboratory tests revealed severe hypothyroidism, hypoalbuminemia, and elevated serum IgG-4 levels. Upper and lower endoscopies showed diffuse gastric and colonic polyposis with chronic inflammation, eosinophilic infiltration, and hyperplastic changes. Diagnosis of CCS was confirmed. Treatment included corticosteroids and thyroid hormone replacement.

**Conclusion:** This case highlights CCS diagnostic challenges, particularly when misdiagnosed as inflammatory bowel disease. The coexistence of hypothyroidism and elevated serum IgG-4 levels suggests a possible autoimmune component. Early recognition of this rare syndrome is essential for appropriate management.

## 1. Introduction

Cronkhite–Canada syndrome (CCS) is a rare, nonhereditary gastrointestinal polyposis syndrome first described in 1955 by Cronkhite and Canada [1]. Since then, only around 500 cases have been described, mainly in Japan, posing diagnostic and therapeutic challenges. The syndrome is characterized by diffuse gastrointestinal polyps, alopecia, onychodystrophy, and hyperpigmentation, presenting with debilitating symptoms such as diarrhea, malabsorption, and protein-losing enteropathy. Despite its rarity, management of CCS has markedly improved with research efforts that have allowed disease understanding and management [2]. Hypothyroidism, while not typical of CCS, has been sporadically reported in association with the syndrome [3–7]. Associations with autoimmune diseases have been described, like rheumatoid arthritis, suggesting a link between endocrine and immune dysfunction and the pathophysiology of CCS [2]. Treatment includes corticosteroids as a first line option, though other immunosuppressants such as azathioprine have been trialed, alongside nutritional support to correct protein-losing enteropathy [2]. Here, we present a unique case of CCS accompanied by primary hypothyroidism and elevated serum IgG-4 levels. This case underlines the importance of a multidisciplinary approach in addressing such complex manifestations while contributing to the limited literature on its association with thyroid dysfunction and possible autoimmunity.

## 2. Case Report

We report a case of a Caucasian male in his late 70s presenting with a 1-month history of dysgeusia, asthenia, and a 4-month history of unexplained weight loss (15 kg) at the time of admission to our Internal Medicine unit. Under the guidance of his general practitioner, he tried antimycotic oral washes for the dysgeusia, without success.

The patient's past medical history revealed the onset of diarrhea 3 years prior, which led to a diagnosis of Crohn's disease after a colonoscopy with multiple biopsies. The exam showed erythematous and edematous mucosa throughout the colon, with multiple pseudopolyps. Pathology report described chronic pancolonic inflammation with abundant eosinophils and focal activity. Furthermore, nine sessile conventional adenomatous polyps were resected, eight of which with tubular histology and low-grade dysplasia, and only one with tubulovillous histology and low-grade dysplasia. A course of mesalazine and budesonide was initiated but shortly after terminated by the patient himself. Upper GI endoscopy was not performed at the time, as the diagnosis of Crohn's disease was considered conclusive.

Past medical history also included diabetes mellitus type 2, chronic obstructive pulmonary disease, arterial hypertension, mild valvular insufficiencies (aortic, tricuspid, and mitral), dyslipidemia, benign prostatic hyperplasia, stage G3b chronic kidney disease, hyperuricemia, and hyperparathyroidism. He was an active smoker (30 packs/year) and seldom consumed alcoholic beverages. His BMI was 22.5 kg/m^2^. He was taking ezetimibe, allopurinol, acetylsalicylic acid, losartan, nebivolol, linagliptin, and pantoprazole. This plethora of comorbidities and important risk factors posed diagnostic and therapeutic challenges, particularly as we had to consider iatrogenic causes of presenting symptoms, as well as any Crohn's disease complications.

Physical examination revealed onychodystrophy of both hands and feet, hyperpigmentation of the hands and face, and bilateral pitting edema of the legs. However, he did not present with alopecia, a typical finding in CCS patients. Laboratory test results showed hemoglobin of 13.8 g/dL, low total protein levels (5.2 g/dL), albumin levels of 2.55 g/dL, TSH of 230 mcg/L, FT3 of 1.38 ng/L, very low levels of FT4 (< 0.42 ng/dL), C-reactive protein of 1.03 mg/dL (reference range: 0–0.5 mg/dL), and creatinine of 2.33 mg/dL. To assess whether malnutrition and malabsorption were the causes of weight loss, we also obtained antitissue transglutaminase antibodies, which were negative, and a nutritional panel including vitamin B12, folates, zinc, ferritin, and vitamin D—only the latter resulting low. Thus, we initiated vitamin D supplementation at 50.000 UI twice a month.

Based on these results, we obtained a thyroid ultrasound as well as a panel of antithyroglobulin binding antibodies (anti-TgAb), anti-TSH-receptor stimulating antibodies (anti-TRAb), and antithyroid peroxidase antibodies (anti-TPO), all of which resulted negative. We requested IgG subclass dosing to explain a possible autoimmune mechanism, which were positive for IgG-4 at 151.26 mg/dL (reference range: 3.92–86.4 mg/dL). A thyroid ultrasound did not show nodules but described a mildly hypoechogenic and heterogeneous parenchyma.

Concomitantly, the patient underwent a colonoscopy to monitor Crohn's disease activity, and a gastroscopy, to assess for any anomalies that could explain dysgeusia.

**Figure 1:**
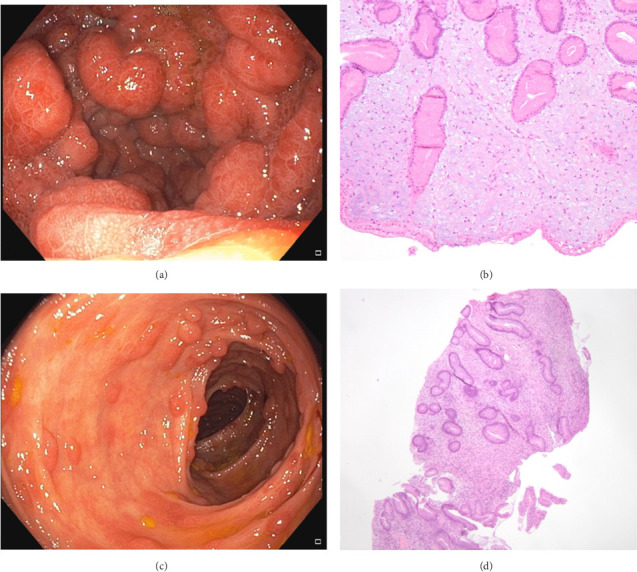
(a) Esophagogastroduodenoscopy showing diffusely erythematous and hyperplastic appearance of the gastric mucosa. (b) Bioptic samples of gastric corpus showing an atrophic oxyntic mucosa characterized by tortuous, irregular and occasionally cystic glands and expanded, edematous lamina propria (hematoxylin and eosin stain, magnification ×10). (c) Colonoscopy showing numerous sessile polyps and slightly erythematous mucosa of the colon. (d) Vaguely polypoid bioptic samples of large bowel mucosa showing elongated nondysplastic glands with occasional cystic dilated crypts and edematous lamina propria comprising mononuclear cells and eosinophils (hematoxylin and eosin stain, magnification ×4).

Gastroscopy revealed a diffusely erythematous and hypertrophic appearance of gastric mucosa (Figure 1(a)). The duodenum appeared erythematous as well, with a papular-like mucosa. Pathology report of gastric mucosa showed mild chronic inflammation, striking foveolar hyperplasia, and marked edema of lamina propria (Figure 1(b)), findings that are in line with those from the colonoscopy. No histologic alterations were detected in duodenal biopsies.

Colonoscopy revealed diffuse adenomatous and hamartomatous-like sessile polyps, some of which were confluent, throughout the colon (Figure 1(c)). The distal ileum was also erythematous and edematous. Resecting polyps was not feasible given their numerosity, but multiple biopsies were performed. Pathology report showed mild active inflammation with an eosinophilic component, expansion of the lamina propria, and hyperplastic features of the glandular epithelium, with occasional markedly dilated glands (Figure 1(d)). Only few biopsied polyps revealed conventional adenomas, tubular type, with low-grade dysplasia.

Our patient satisfied all major diagnostic criteria for CCS amongst those proposed by Hokari et al. [8], even in the absence of alopecia, thus the previous diagnosis of Crohn's disease was revoked and definitive diagnoses of CCS and hypothyroidism were made. On this occasion, we obtained verbal informed consent to publish this case report. Furthermore, at the time of diagnosis, the clinical severity was severe, scoring 11 points.

The case was discussed within our Internal Medicine and Gastroenterology teams, and we decided to start with 50 mg of prednisone and 50 mcg of levothyroxine, both daily. Given our patient's compromised renal function, we decided against aggressive protein supplementation, opting for an oral amino acid supplement to take once daily. Treatment outcomes could not be assessed as the patient died shortly after because of a severe episode of acute heart failure complicated by pneumonia.

## 3. Conclusions

We hereby report a rare case of CCS with concomitant hypothyroidism and multiple comorbidities. Diagnosing CCS is particularly challenging when the gastrointestinal tract is selectively investigated and not evaluated as a whole, leaving room for confounding histological findings for an inflammatory bowel disease, as was the case for our patient. We hope to spread awareness regarding this rare syndrome and its management.

## Data Availability

The data that support the findings of this study are available on request from the corresponding author. The data are not publicly available due to privacy or ethical restrictions.
